# Efficacy of lower arch leveling, lower incisors’ root resorption, and pain associated with the correction of curve of Spee using different orthodontic archwires: a randomized clinical trial

**DOI:** 10.1007/s00784-022-04672-x

**Published:** 2022-08-23

**Authors:** Yousef H. Nasrawi, Elham S. Abu Alhaija, Emad F. Al Maaitah

**Affiliations:** 1 Faculty of Dentistry, Division of Orthodontics, Department of Preventive Dentistry, Jordan University of Science and Technology, P.O. Box 3030, Irbid, Jordan; 2 College of Dental Medicine, QU Health, Qatar University, P.O. Box 2713, Doha, Qatar

**Keywords:** Curve of Spee, Archwires, Root resorption, Pain

## Abstract

**Objectives:**

To compare between 3 archwires (AWs) for leveling curve of Spee (COS) in terms of efficacy of reduction, external apical root resorption (EARR), pain experienced, and the lower arch dimensional changes during COS leveling.

**Trial design:**

Randomized clinical trial.

**Setting:**

Jordan University of Science and Technology Postgraduate dental clinics.

**Material and methods:**

Fifty-three subjects with COS > 5 mm were included in this study. The subjects were randomly divided into three groups based on the AW used: group 1, 0.017 × 0.025-inch stainless-steel (SS) AW; group 2, 0.019 × 0.025-inch SS AW; and group 3, 0.021 × 0.025-inch β-titanium (TMA) AW. The intervention was randomly allocated using the permuted random block size of 3 with a 1:1:1 allocation ratio. In the three groups, a 5-mm depth reverse COS was placed in the AWs. The following time points were defined for COS assessment: T1, before interventional leveling AW placement; and T2–T7, 1–6 months after interventional leveling AW placement. Records consisted of dental study models and periapical (PA) radiographs. Pain scores were recorded using visual analogue scale. Patients were followed up on a monthly basis until COS < 1.5 mm.

**Main outcome measures:**

COS depth reduction, lower incisors’ EARR, pain scores, and arch dimensional changes.

**Results:**

An overall reduction of 3.82 mm, 4.47 mm, and 3.85 mm of the depth of COS was achieved in groups 1, 2, and 3, respectively. The mean differences of 0.65 mm between groups 1 and 2 and 0.62 mm between groups 2 and 3 were significant at *P* < 0.05. Lower incisors’ EARR during leveling COS ranged from 0.68 to 0.72 mm, from 0.63 to 0.82 mm, and from 0.53 to 0.88 mm in groups 1, 2, and 3, respectively (*P* > 0.05). Higher pain scores were reported by group 2 subjects during the first 24 h. Arch length and width increased significantly in groups 2 and 3 (*P* < 0.05). In all groups, COS leveling was achieved by lower incisor intrusion and proclination and lower molar extrusion.

**Conclusions:**

All investigated AWs were effective in leveling COS with minimal lower incisors’ EARR (< 1 mm). COS was leveled by lower incisors’ intrusion and proclination and lower molar extrusion. Pain scores were the highest in group 2 during the first 24 h.

**Clinical relevance:**

The 3 investigated leveling AWs were effective for the leveling COS and at the same time safe on the roots of the lower anterior teeth.

## Introduction

In modern orthodontics, the curve of Spee (COS) refers to the natural progression upwards of the teeth curvature from the incisors through the premolars and molars. The leveling of teeth during orthodontic treatment involves bringing the incisal edges of the anterior teeth and the buccal cusps of the posterior teeth into a horizontal plane level [1].

A deep COS is usually associated with an increased overbite. COS is most severe in Class II division 2 subjects, followed by Class II division 1 subjects and Class I subjects, with the least amount of depth in Class III subject [2]. Paes-Souza et al. [3] conducted a systematic review with meta-analysis to evaluate the variations in the depth of COS based on the different dentoskeletal characteristics. They concluded that dentoskeletal Class II, Class III malocclusion, deep bite, and the hypodivergent skeletal pattern affected the depth of the COS. Anyway, they suggested that definitive conclusions are not possible due to the very low certainty of the evidence.

Three possible ways to level out a lower arch with excessive COS [4] are absolute intrusion of lower incisors, relative intrusion (keeping incisors where they are while allowing posterior teeth to erupt), and extrusion of posterior teeth.

Leveling COS by extrusion is usually accomplished with the use of continuous archwires (AWs), by placing a reverse COS in the mandibular arch. The correction is usually achieved by premolar extrusion with little incisor intrusion [5]. Parker et al. [6] stated that the correction of the overbite occurred by proclination of lower incisors and extrusion of lower molars. Bernstein et al. [7] reported that leveling the COS with continuous AW takes place by a combination of premolar extrusion and, to a lesser extent, incisor intrusion.

Various methods have been used to quantify the COS. These methods range from the common two-dimensional (2D) approaches measuring either directly from dental casts [8, 9] or digitized images to the beginning of three-dimensional (3D) analysis [10]. COS is usually measured as the sum of the right and left side maximum depths of the COS to a reference line from the central incisors to distal cusp tips of second molars divided by 2.

External apical root resorption (EARR) is an undesirable side effect in orthodontic treatment, and it has a multifactorial etiology [11]. It is estimated that up to 90% of orthodontically treated teeth have some extent of external apical root resorption, and up to 15% of these cases show severe EARR of more than 4 mm [12]. Chiqueto et al. [13] reported a statistically significant correlation between root resorption, the amount of deep bite reduction, and the amount of maxillary incisor intrusion. However, Costopoulos and Nanda [14] found a weak correlation between root resorption and incisors’ intrusion.

Pain has been stated as a factor that reduces patient compliance during treatment and a reason that patients discontinue treatment or miss appointments [15, 16]. It has been reported that between 87 and 95% of adolescents experienced pain during the first 24 h of fixed orthodontic treatment [15].

Up to this day, many orthodontists are using a reverse COS continuous AW for mandibular arch leveling without clear evidence which is the most effective rectangular AW’s size to be used. Also, the effect of using continuous rectangular AW on lower incisors’ root resorption and patient’s pain perception has not been studied before. Therefore, this investigation was carried out to investigate the efficacy of 3 different dimensions of continuous leveling AWs (0.017 × 0.025-inch stainless steel (SS), 0.019 × 0.025-inch SS, and 0.021 × 0.025-inch beta titanium (TMA)) for the correction of excessive COS in the mandibular arch and to report on lower incisors’ EARR, incisor intrusion, pain scores, and arch dimensional changes associated with leveling COS using the above AWs.

## Material and method

### Study design

This study was a randomized clinical trial with a 1:1:1 allocation ratio. The methods were not changed after trial initiation.

### Participants, eligibility criteria, and settings

The study was reviewed and approved by the Institutional Review Board at Jordan University of Science and Technology (JUST) (approval number 78/117/2018). This trial was registered at ClinicalTrials.gov with identifier number NCT04549948. The participants for this study were recruited from patients attending orthodontic clinics at the postgraduate dental clinics/Jordan University of Science and Technology. Orthodontic treatment then was carried out at the postgraduate dental clinics/Jordan University of Science and Technology. Study model analysis was performed at the postgraduate dental teaching laboratory/Jordan University of Science and Technology. All subjects who agreed to participate in the study signed a consent form for participation after clarifying the purpose of the intervention.

A total of 60 subjects who fulfilled the inclusion criteria were invited to participate in the study. The inclusion and exclusion criteria for this study are shown in Table 1.

**Table 1:** Inclusion and exclusion criteria

Inclusion criteria
Age ≥ 16 years and ≤ 30 years
Normally inclined or retroclined lower incisors
Presence of deep bite
Depth of curve of Spee ≥ 5 mm
Mild lower arch crowding < 4 mm
Non-extraction treatment plan
Averaged or reduced lower vertical height
Good oral hygiene and healthy periodontium
All permanent teeth are present except for the third molars
Exclusion criteria
Severe crowding in lower arch where extraction treatment is indicated
Missing permanent teeth other than third molars
Poor oral hygiene and presence of periodontal disease
Presence of medical condition or being under medication that could affect the treatment
History of previous orthodontic treatment
Smoking

### Sample size

Sample size was calculated using the G*power 3.1.9 program [17]. Univariate analysis revealed significant variability between subjects (*F* = 4.45, *P* = 0.017, partial eta squared = 0.15). The estimate was based on a study by Rozzi et al. [18] who evaluated leveling of the CoS in different skeletal vertical patterns. They reported a mean of 2.69 ± 1.90 mm and 2.34 ± 1.65 mm of CoS correction in reduced and average skeletal vertical groups, respectively. Assuming a small effect size difference (0.2) between groups, power analysis yielded a total sample size estimate of 51 subjects (17 patients per group) at a conventional alpha level (0.05) and desired power (1 – *β*) of 0.85. To build up for attrition rate of 10%, initial recruitment targeted a total of 57 subjects (19 patients/group). A small effect size difference (0.2) was assumed in order to detect the small differences between groups.

### Randomization

After recruiting patients who met the inclusion criteria and just before the insertion of the leveling AWs, the intervention was randomly allocated using the permuted random block size of 3 with a 1:1:1 allocation ratio by one research assistant. The allocation sequence was concealed from the researcher (Y.N.) by sequentially numbered, opaque, sealed, and stapled envelopes before the intervention. Patients were then asked to pick a sealed envelope to assign the method of intervention.

### Blinding

The patients were blinded to the intervention used. However, it was not possible to blind the clinician during treatment. Measurements of the dental casts were performed by one dental assistant (N.G) and measurements of lower anterior teeth root resorption and cephalometric superimposition were performed by one dental clinician (K.G) who were blinded to the type of the intervention used.

### Intervention

All subjects were treated by the same orthodontic resident (Y.N.) using pre-adjusted edgewise fixed appliance on upper and lower arches without extraction (American Orthodontics, 0.022 × 0.028-inch Roth prescription brackets). All AWs were ovoid in shape from 3 M Unetik company (Monrovia, Calif). A standardized bonding technique was applied according to the manufacturer’s instructions and vertical bracket positioning was done using bracket gauge (4 mm from incisal tip for incisors, 4.5 mm from occlusal tip for canines and premolars). Pre-treatment (T0) records (lateral cephalogram (LC), orthopantomogram (OPG), and study casts) were taken for all patients.

At the bond-up visit, and because of the deep bite, glass ionomer (G.I) posterior bite blocks were added on the upper second molars to raise the bite and allow lower arch bond up at the same visit. G.I blocks were kept during early alignment stage and removed before the placement of interventional reverse COS leveling AWs. Teeth alignment started with round 0.016-inch NiTi AW which included the upper and lower second molars, and then a sequence of 0.018-inch NiTi and 0.016 × 0.022-inch NiTi was inserted before 0.017 × 0.025-inch NiTi AWs were reached. The appointment visits were the same for all patients during the intervention (every 4 weeks).

After alignment (average duration 5.51 ± 0.61 months) and before the insertion of the reverse COS leveling AWs, LC (LC1), periapical PA (PA1) radiographs for lower incisors, and alginate impression for lower arch were taken for all patients at this time point (T1).

Of the total subjects, 2 patients had their COS leveled to a less than 3 mm during alignment stage. Accordingly, they were excluded from the trial before intervention started. None of the included subjects had their COS leveled before trial period (6 months).

Afterwards, and based on their allocation group, 3 different leveling continuous AWs were inserted as follows:

Group 1: Leveling of COS using 0.017 × 0.025-inch SS AW (20 patients; 13 females, 7 males)

Group 2: Leveling of COS using 0.019 × 0.025-inch SS AW (19 patients; 14 females, 5 males)

Group 3: Leveling of COS using 0.021 × 0.025-inch TMA AW (19 patients; 15 females, 4 males)

In the 3 studied groups, a 5-mm depth reversed COS was placed in the interventional AWs using tweed plier distal to lower canines. Measurement of the applied reverse COS in the AWs was done using digital caliper and inserted without a cinch back. The anterior labial crown torque was removed from all AWs by holding the AW mesial to the first premolars with a pair of tweed pliers and “twisting” the AW to achieve a zero torque “flat” surface anteriorly. This was further checked by holding the tweed pliers at the anterior and posterior segments of the AW and observing the lack of torque anteriorly. All teeth were included in the fixed orthodontic appliance including lower second molars. Patients were instructed to contact the clinic within 24 h if any bracket were debonded. The following time points were defined: T1, before placement of interventional leveling AW; and T2–T7, 1 month, 2 months, 3 months, 4 months, 5 months, and 6 months after placement of the leveling AWs.

On each monthly visit, AWs were checked to ensure absence of any deformation and alginate impression was taken for the lower arch. Utility wax was used to cover the lower arch brackets not to distort the impression upon removal from the mouth. Dental casts were poured the same day in the laboratory using dental gypsum type II. At T7, PA radiographs (PA2) for lower anterior teeth and pre-finish LC (LC2) were taken for all subjects.

### Outcomes

#### Primary outcome

## Depth of COS

The depth of COS was measured manually using a digital caliper as the perpendicular distance between the deepest cusp tip and a flat plane that was laid on top of the mandibular dental cast, touching the incisal edges of the central incisors and the distal cusp tips of the second molars [9]. It was measured on the right and left sides of the mandibular arch and the average value was included in the analysis. All dental casts were trimmed and mounted equally on a dental surveyor to ensure accurate results.

### Secondary outcome

## Lower incisors’ EARR (Fig. 1)

**Fig. 1:**
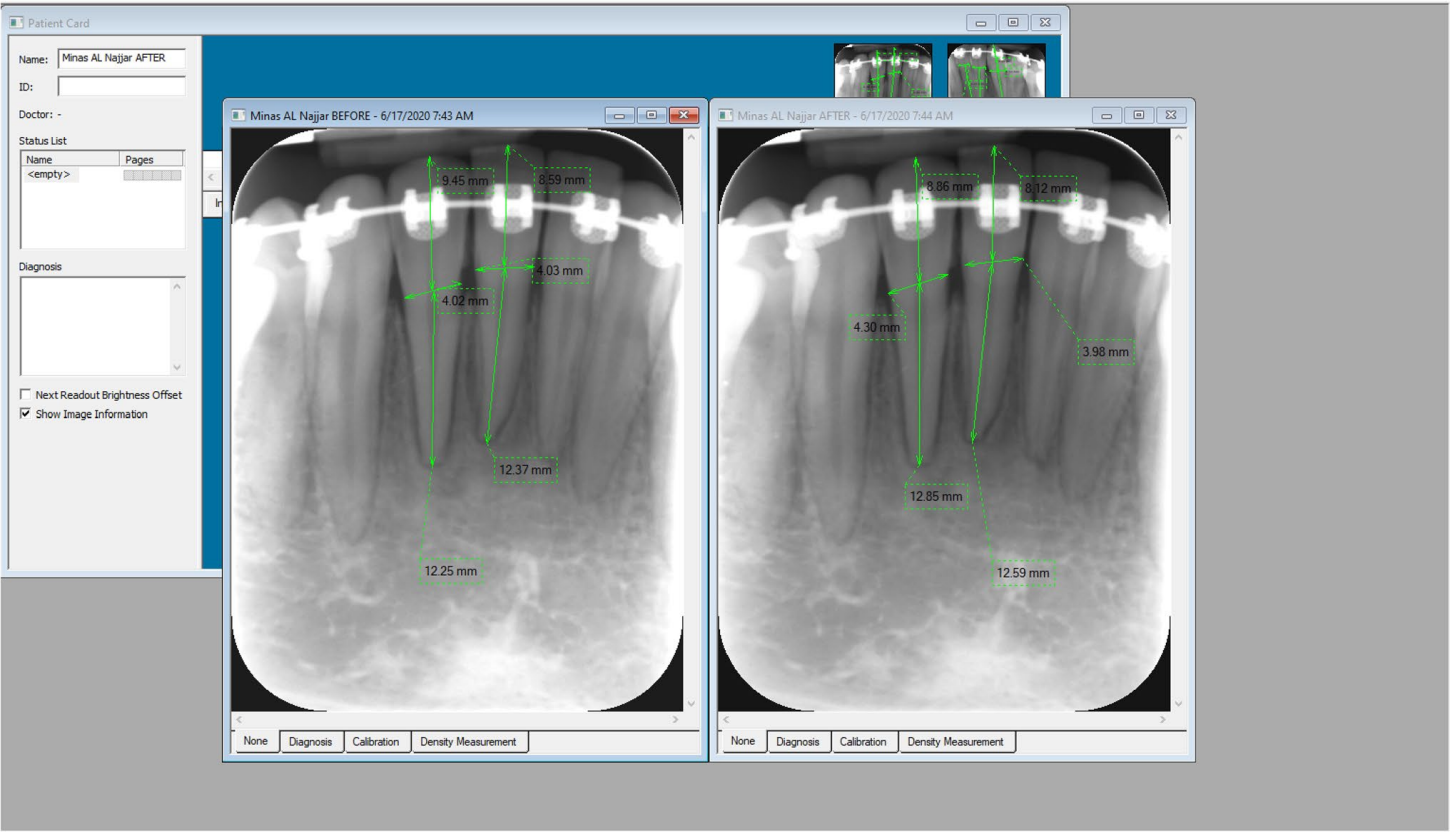
Example of using DIGORA for Windows 2.5 to measure crown and root length before treatment and after placement of leveling AW

Digital PA radiographs were taken for the lower incisors for each patient by applying the paralleling technique using film holders and intraoral sensors from KAVO Company. Those sensors were scanned using a DIGORA™ Optime scanner and a digital version of the radiograph was imported from the scanner to the PC using DIGORA for Windows 2.5 software. For each patient, 2 digital PA radiographs were taken: at T1 (PA1) and at T7 (PA2). Crown length was measured from the initial (C1) and the final (C2) PA radiographs, as a linear distance from the median line of cemento-enamel junction to the incisal tip. Root length was measured from the median line of cemento-enamel junction to the tip of the root apex in both initial (R1) and final (R2) radiographs as suggested by Linge and Linge [19]. A correction factor for magnification between the start (T1) and final (T7) radiographs was calculated as C1/C2. Apical root resorption was measured as the difference between root length at T1 (R1) and T7 (R2) multiplied by the correction factor: apical root resorption = R1–R2 × (C1/C2). Image resolution for each radiograph was calibrated to 15.63 pixels/mm in both horizontal and vertical dimensions to achieve the most accurate measurements. Root/crown (R/C) ratio before orthodontic alignment was measured from the pre-treatment OPG (T0), whereas it was measured at T1 and T7 from the PA (PA1, PA2) images.

## Perception of pain

Pain was assessed over the first week after the insertion of the reversed COS AWs by means of a 10-point visual analogue scale (VAS) of 10 cm length. Maximum subjective pain experienced by each patient was recorded: 1 h, 24 h, 48 h, and 1 week after the insertion of interventional AW. A recording sheet with visual analogue scales was given to all patients with verbal instructions on how to complete the VAS questionnaire by marking a point on the 10-cm line which they believed to best represent the maximum pain they experienced that day, 0 indicates no pain and 10 indicates intolerable pain.

## Lower arch dimensions

### Arch length (AL)

It is the distance between the mid-incisal edge and the midpoint of a line joining the distobuccal cusps of the first molars.

### Intercanine width (ICW)

It is the distance between the cusp tip of the right canine and the cusp tip of the left canine in the lower arch.

### Intermolar width (IMW)

It is the distance between the mesio-buccal cusp tip of the right first molar and the mesio-buccal cusp tip of the left first molar in the lower arch.

## Incisor and molar vertical change

Mandibular superimposition was performed using the internal cortical outline of the symphysis. Pre-leveling LC (LC1) was placed on the graphic tablet of the digitizing system over a millimeter-graded sheet. The pre-finish LC (LC2) was superimposed on LC1. The difference between every related point was measured by calculating the number of squares (each square on the graded sheet equal 1 mm). Vertical changes in root and crown positions of lower central incisor and lower first molars were registered [20].

### Interim analyses and stopping guidelines

Not applicable.

### Method error

Ten randomly selected dental casts and PA radiographs were re-measured after 2-week interval by the same investigator and the intra-observer reliability was calculated using Houston’s coefficient of reliability.

### Statistical analysis

Statistical analysis was performed with the use of the Statistical Package for Social Science (SPSS) computer software (SPSS 28, SPSS Inc., NY, USA). Descriptive statistics were calculated for all the measured variables for each group. Intention to treat (ITT) analysis was performed. The Shapiro–Wilk test to assess normality of numeric data indicated that only EARR data was normally distributed. The non-parametric Wilcoxon test was applied to detect within-group differences in the measured parameters at the different time points and the Mann–Whitney *U* test was used to detect differences between the studied groups. One-way ANOVA was used to detect EARR differences between the studied groups. *P* value was set at 0.05 level.

## Results

Houston’s coefficient of reliability was above 0.94 for the measured variables.

### Subjects (Fig. 2)

**Fig. 2:**
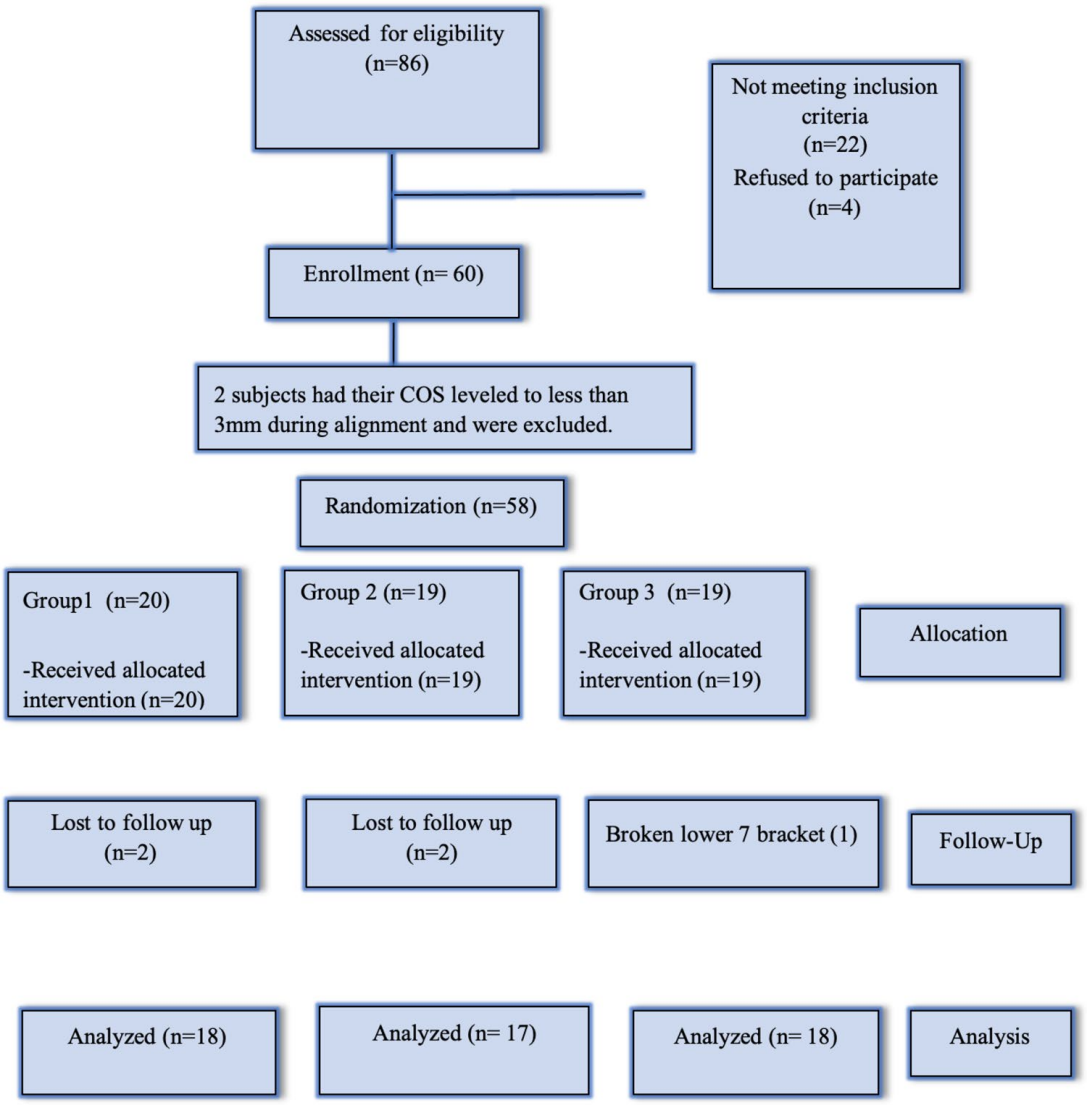
CONSORT flow chart showing patients’ flow during the trial

Subjects were recruited between December 2018 and September 2019, with the final data collection in June 2020. Initially, 60 subjects participated in the study. Of the total subjects, 2 patients had their COS leveled to a less than 3 mm during alignment stage. Accordingly, they were excluded from the trial before intervention started. Fifty-eight subjects received the planned intervention. No bracket or tube detachment was reported during the alignment stage. However, during the leveling stage, 5 patients (2 in group 1 and 3 in group 3) had broken molar tubes which were replaced within 24 h. In group 1, 20 patients had their COS leveled using 0.017 × 0.025-inch SS AW with a reverse COS. Two patients were excluded from final analysis (missed their appointments). In group 2, 19 patients had their COS leveled using 0.019 × 0.025-inch SS AW with a reverse COS. Two patients were excluded from final analysis (missed their appointments). In group 3, 19 patients had their COS leveled using 0.0215 × 0.025-inch TMA AW with a reverse COS. One patient was excluded from final analysis (broken lower second molar tube which was not reported within 24 h).

During the analysis stage, there were records for 53 patients (40 females and 13 males): 18 patients in group 1 (12 females and 6 males), 17 patients in group 2 (12 females and 5 males), and 18 patients in group 3 (15 females and 3 males). The end point of this study was to 6 months after the intervention. Baseline data for the included subjects are shown in Table 2.

**Table 2:** Baseline data for the subjects included in the study

	Pre-treatment (T0)			Just before intervention (T1)		
	Group 1 (*n* = 20) Mean (SD)	Group 2 (*n* = 19) Mean (SD)	Group 3 (*n* = 19) Mean (SD)	Group 1 (*n* = 20) Mean (SD)	Group 2 (*n* = 19) Mean (SD)	Group 3 (*n* = 19) Mean (SD)
Sella-Nasion-point A (SNA) °	82.7 (4.7)	83.2 (2.23)	82.1 (1.92)	83.0 (3.14)	84.0 (1.66)	82.7 (2.08)
Sella-Nasion-point B (SNB)°	77.6 (3.21)	77.9 (2.66)	77.6 (2.31)	78.2 (2.31)	78.2 (1.94)	78.0 (1.30)
Point A-Nasion-point B (ANB)°	4.9 (0.52)	5.1 (0.43)	5.0 (0.97)	4.7 (1.71)	5.2 (0.86)	5.0 (1.35)
Maxillary/mandibular (MM) plane °	23.8 (4.80)	24.7 (1.29)	22.5 (1.82)	24.7 (4.80)	25.0 (5.18)	22.9 (2.43)
Gonial angle °	126.21 (5.01)	124.53 (4.50)	125.21 (4.34)	127.36 (4.40)	125.73 (4.06)	126.43 (3.63)
Anterior facial height (mm)	119.71 (6.52)	118.13 (5.51)	122.29 (5.68)	121.07 (6.39)	119.47 (4.91)	123.36 (5.08)
Posterior facial height (mm)	75.29 (3.79)	73.13 (2.80)	74.50 (3.41)	76.21 (3.53)	74.40 (2.41)	75.57 (3.08)
Ramal height (mm)	52.57 (4.78)	51.20 (6.49)	51.93 (6.74)	52.57 (4.78)	51.33 (6.39)	52.07 (6.67)
Lower incisor-point A/pogonion (mm)	− 1.30 (0.88)	− 1.12 (1.42)	− 1.05 (1.78)	− 1.21 (1.75)	− 0.93 (1.43)	− 0.86 (1.69)
Lower incisors-mandibular plane °	91.2 (1.99)	88.1 (1.46)	91.3 (2.38)	95.7 (4.09)	90.9 (3.51)	94.3 (3.62)
Upper incisors-maxillary plane °	98.4 (0.64)	97.5 (1.32)	99.4 (1.96)	103.8 (0.56)	101.6 (1.94)	103.6 (2.15)
Inter-incisal angle °	131.7 (4.21)	139.3 (2.57)	139.4 (2.18)	132.3 (6.41)	141.3 (3.97)	136.0 (4.16)
Overbite (mm)	5.6 (1.33)	5.8 (0.32)	4.8 (2.87)	4.9 (0.77)	5.3 (1.48)	4.2 (1.35)
Overjet (mm)	1.7 (1.0)	2.9 (1.0)	2.5 (1.0)	2.1 (0.97)	3.2 (1.0)	3.1 (1.0)
Curve of Spee (mm)	5.35 (0.31)	6.26 (0.11)	5.98 (0.48)	5.11 (0.58)*	5.59 (0.62)*	5.22 (0.73)
Lower arch crowding (mm)	2.8 (0.9)*	2.1 (0.4)*	2.6 (1.2)			
Age (years)	20.93 (3.37)	22.47 (4.06)	20.0 (2.10)			

^*^P < 0.05

### Numbers analyzed at each time point

First month after starting in the intervention (T2), one patient from groups 1 and 2 were excluded from the analysis (*n* = 19 in group 1, *n* = 18 in group 2, and *n* = 19 in group 3). At T3, 3 patients (one patient from each group) were excluded from the analysis (*n* = 18 in group 1, *n* = 17 in group 2, and *n* = 18 in group 3). None of the subjects was excluded from the analysis from T4 to T7.

### Leveling of COS (Table 3)

**Table 3:** Means and SDs for the depth of COS (mm), Wilcoxon signed-rank (within-group differences) and Mann–Whitney *U* (between-group differences) standardized test statistics and *P* values in the 3 studied groups

	T1	T2	T3	T4	T5	T6	T7	T1–T7
	Mean (SD)	Mean (SD)	Mean (SD)	Mean (SD)	Mean (SD)	Mean (SD)	Mean (SD)	Mean (SD)
Group 1 (0.017 × 0.025 SS)	5.11 (0.58)	4.42 (0.49)	3.83 (0.38)	3.22 (0.39)	2.97 (0.50)	1.29 (0.44)	0.67 (0.42)	3.82 (0.70)
Group 2 (0.019 × 0.025 SS)	5.59 (0.62)	4.77 (0.56)	4.12 (0.49)	3.35 (0.61)	2.82 (0.53)	1.82 (0.50)	1.12 (0.33)	4.47 (0.51)
Group 3 (0.021 × 0.025 TMA)	5.22 (0.73)	4.61 (0.78)	4.06 (0.54)	3.78 (0.43)	3.22 (0.43)	2.39 (0.50)	1.39 (0.52)	3.85 (0.78)
Within-group differences in COS depth changes at different time points (mm) (related samples Wilcoxon signed-rank test)								
	COS changes T1–T2 (standardized test statistics, *P* value)	COS changes T2–T3 (standardized test statistics, *P* value)	COS changes T3–T4 (standardized test statistics, *P* value)	COS changes T4–T5 (standardized test statistics, *P* value)	COS changes T5–T6 (standardized test statistics, *P* value)	COS changes T6–T7 (standardized test statistics, *P* value)	COS changes T1–T7 (standardized test statistics, *P* value)	
Group 1	(− 3.42, *P* < 0.001)	(− 3.21, *P* = 0.001)	(− 3.28, *P* = 0.001)	(− 2.25, *P* = 0.024)	(− 3.77, *P* < 0.001)	(− 3.94, *P* < 0.001)	(− 3.77, *P* < 0.001)	
Group 2	(− 3.49, *P* < 0.001)	(− 3.46, *P* < 0.001)	(− 3.61, *P* < 0.001)	(− 3.00, *P* = 0.003)	(− 3.82, *P* < 0.001)	(− 3.42, *P* < 0.001)	(− 3.73, *P* < 0.001)	
Group 3	(− 3.32, *P* < 0.001)	(− 2.33, *P* = 0.020)	(− 2.24, *P* = 0.025)	(− 3.16, *P* = 0.002)	(− 3.87, *P* < 0.001)	(− 3.90, *P* < 0.001)	(− 3.83, *P* < 0.001)	
Between-group differences (Mann–Whitney *U* test)								
	COS changes T1–T2 (standardized test statistics, *P* value)	COS changes T2–T3 (standardized test statistics, *P* value)	COS changes T3–T4 (standardized test statistics, *P* value)	COS changes T4–T5 (standardized test statistics, *P* value)	COS changes T5–T6 (standardized test statistics, *P* value)	COS changes T6–T7 (standardized test statistics, *P* value)	COS changes T1–T7 (standardized test statistics, *P* value)	
Groups 1 and 2	(− 0.63, *P* = 0.590)	(− 0.34, *P* = 0.775)	(− 1.11, *P* = 0.369)	(− 1.63, *P* = 0.153)	(− 1.82, *P* = 0.207)	(1.31, *P* = 0.318)	(2.79, *P* = 0.009)	
Groups 1 and 3	(− 0.33, *P* = 0.791)	(− 0.40, *P* = 0719)	(− 2.08, *P* = 0.068)	(− 1.83, *P* = 0.111)	(− 0.35, *P* = 0.815)	(− 1.46, *P* = 0.293)	(0.33, *P* = 0.767)	
Groups 2 and 3	(− 0.98, *P* = 0.405)	(− 0.59, *P* = 0.599)	(− 2.84, *P* = 0.013)	(− 0.15, *P* = 0.909)	(− 1.59, *P* = 0.303)	(− 2.31, *P* = 0.057)	(2.53, *P* = 0.022)	

*T1*, before inserting interventional arch wires with reverse COS; *T2*, 1 month; *T3*, 2 months; *T4*, 3 months; *T5*, 4 months; *T6*, 5 months; *T7*, 6 months after interventional arch wire insertion

After 6 months of lower arch leveling, the depth of CoS was reduced to 0.67 mm, 1.12 mm, and 1.39 mm COS in groups 1, 2, and 3, respectively. Significant monthly reduction of the depth of the CoS was achieved in all groups (*P* < 0.05). Group 2 (0.019 × 0.025 SS) produced more CoS reduction compared to group 1 (0.017 × 0.025 SS) (*P* < 0.01) and group 3 (0.021 × 0.025 TMA) (*P* < 0.05).

### Lower anterior teeth root resorption (Table 4)

**Table 4:** Means, SDs, and Diff. between the means of EARR (mm) and C/R ratio, SE of the mean differences, 95% C.I., and *P* value for the 3 studied groups at different time intervals

Variables	Group 1	Group 2	Group 3	Groups 1 and 2		Groups 1 and 3		Groups 2 and 3	
	Mean (SD)	Mean (SD)	Mean (SD)	Mean diff (SE, *P* value)	95% C.I	Mean diff (SE, *P* value)	95% C.I	Mean Diff (SE, *P* value)	95% C.I
EARR									
EARR 32	0.68 (0.22)	0.61 (0.35)	0.67 (0.41)	0.07 (0.12, *P* = 0.587)	− 0.18–0.32	0.01 (0.12, *P* = 0.905)	− 0.24–0.27	− 0.06 (0.12, *P* = 0.673)	− 0.31–0.20
EARR 31	0.66 (0.17)	0.52 (0.29)	0.46 (0.35)	0.14 (0.10, *P* = 0.193)	− 0.07–0.35	0.20 (0.10, *P* = 0.068)	− 0.02–0.41	− 0.06 (0.10, *P* = 0.565)	− 0.15–0.27
EARR 41	0.67 (0.20)	0.44 (0.30)	0.81 (0.46)	0.23 (0.12, *P* = 0.072)	− 0.02–0.48	− 0.14 (0.12, *P* = 0.269)	− 0.40–0.11	− 0.37 (0.12, *P* = 0.005)	− 0.62 to − 0.12
EARR 42	0.76 (0.23)	0.87 (0.61)	0.78 (0.33)	− 0.11 (0.15, *P* = 0.474)	− 0.44–0.21	− 0.02 (0.16, *P* = 0.882)	− 0.35–0.30	0.09 (0.16, *P* = 0.571)	− 0.23–0.41
Root/crown (R/C) ratio									
R/C ratio 32 (T0)	2.05 (0.29)	2.18 (0.28)	2.34 (0.27)	− 0.13 (0.10, *P* = 0.22)	− 0.34–0.08	− 0.29 (0.10, *P* = 0.009)	− 0.51 to − 0.08	− 0.16 (0.10, *P* = 0.129)	− 0.38–0.05
R/C ratio 32 (T1)	2.01 (0.29)	2.15 (0.27)	2.31 (0.27)	− 0.14 (0.10, *P* = 0.186)	− 0.35–0.07	− 0.30 (0.11, *P* = 0.006)	− 0.51 to − 0.09	− 0.16 (0.10, *P* = 0.121)	− 0.37–0.05
R/C ratio 32 (T2)	1.93 (0.29)	2.07 (0.25)	2.22 (0.27)	− 0.14 (0.10, *P* = 0.168)	− 0.35–0.06	− 0.30 (0.10, *P* = 0.006)	− 0.51 to − 0.09	− 0.15 (0.10, *P* = 0.130)	− 0.36–0.04
R/C ratio 31 (T0)	2.11 (0.36)	1.98 (0.35)	2.26 (0.25)	0.13 (0.12, *P* = 0.27)	− 0.11–0.38	− 0.14 (0.12, *P* = 0.249)	− 0.39–0.10	− 0.28 (0.12, *P* = 0.026)	− 0.52 to − 0.03
R/C ratio 31 (T1)	2.08 (0.33)	1.98 (0.34)	2.24 (0.23)	0.10 (0.11, *P* = 0.349)	− 0.12–0.34	− 0.16 (0.12, *P* = 0.178)	− 0.39–0.08	− 0.26 (0.11, *P* = 0.024)	− 0.49 to − 0.04
R/C ratio 31 (T2)	1.99 (0.34)	1.91 (0.31)	2.18 (0.23)	0.09 (0.11, *P* = 0.419)	− 0.13–0.32	− 0.18 (0.11, *P* = 117)	− 0.41–0.05	− 0.27 (0.11, *P* = 0.019)	0.05–0.50
R/C ratio 41 (T0)	2.05 (0.33)	2.17 (0.27)	2.34 (0.33)	− 0.12 (0.11, *P* = 0.318)	− 0.36–0.12	− 0.29 (0.11, *P* = 0.017)	− 0.53 to − 0.06	− 0.17 (0.11, *P* = 0.135)	− 0.41–0.05
R/C ratio 41 (T1)	2.04 (0.32)	2.14 (0.26)	2.27 (0.30)	− 0.11 (0.11, *P* = 0.345)	− 0.33–0.12	− 0.24 (0.11, *P* = 0.041)	− 0.46 to − 0.01	− 0.13 (0.11, *P* = 0.240)	− 0.35–0.09
R/C ratio 41 (T2)	1.94 (0.32)	2.08 (0.26)	2.16 (0.30)	− 0.13 (0.10, *P* = 0.229)	− 0.35–0.09	− 0.21 (0.11, *P* = 0.062)	− 0.44–0.01	− 0.08 (0.10, *P* = 0.471)	− 0.30–0.14
R/C ratio 42 (T0)	2.06 (0.29)	2.19 (0.34)	2.35 (0.23)	− 0.13 (0.11, *P* = 0.240)	− 0.35–0.09	− 0.29 (0.11, *P* = 0.013)	− 0.51 to − 0.06	− 0.15 (0.11, *P* = 0.156)	− 0.38–0.06
R/C ratio 42 (T1)	2.02 (0.30)	2.14 (0.31)	2.33 (0.22)	− 0.12 (0.10, *P* = 0.254)	− 0.33–0.09	− 0.31 (0.11, *P* = 0.006)	− 0.53 to − 0.10	− 0.19 (0.10, *P* = 0.076)	− 0.40–0.02
R/C ratio 42 (T2)	1.93 (0.31)	2.02 (0.28)	2.23 (0.21)	− 0.09 (0.10, *P* = 0.332)	− 0.30–0.10	− 0.30 (0.10, *P* = 0.005)	− 0.51 to − 0.10	− 0.20 (0.10, *P* = 0.048)	− 0.41 to − 0.002

*EARR*, external apical root resorption; *C/R*, crown/root ratio; *T1*, before placement of interventional leveling AW; *T2*, 6 months after placement of the leveling AWs

Lower incisors’ EARR during leveling COS was detected in all studied groups. It ranged from 0.66 to 0.76 mm, from 0.44 to 0.87 mm, and from 0.46 to 0.81 mm in groups 1, 2, and 3, respectively. Lower incisors’ EARR was similar in groups 1 and 2 (*P* > 0.05). Group 3 showed more EARR in the right lower central incisor compared to group 2 (*P* < 0.01).

### Perception of pain (Table 5)

**Table 5:** Means, standard deviations (SDs) of pain scores and pain score changes in the 3 studied groups, Wilcoxon signed-rank (within-group differences) and Mann–Whitney *U* (between-group differences) standardized test statistics, and *P* values in the 3 studied groups

	One hour after insertion of interventional AW (T1)	24 h after insertion of interventional AW (T2)	48 h after insertion of interventional AW (T3)	One week after insertion of interventional AW (T4)
	Mean (SD)	Mean (SD)	Mean (SD)	Mean (SD)
Group 1	3.10 (1.89)	4.65 (1.84)	4.55 (2.12)	2.40 (1.31)
Group 2	3.47 (1.90)	6.16 (1.57)	4.05 (1.65)	2.16 (1.30)
Group 3	3.84 (2.24)	5.63 (2.34)	4.53 (2.14)	2.05 (1.39)
Within-group differences in mean pain score changes at different time points (related samples Wilcoxon signed-rank test)				
	Pain changes T1–T2 (standardized test statistics, *P* value)	Pain changes T2–T3 (standardized test statistics, *P* value)	Pain changes T3–T4 (standardized test statistics, *P* value)	Pain changes T1–T4 (standardized test statistics, *P* value)
Group 1	(2.80, *P* = 0.005)	(− 0.39, *P* = 0.700)	(− 3.40, *P* < 0.000)	(− 1.51, *P* = 0.132)
Group 2	(3.38, *P* < 0.000)	(− 3.76, *P* < 0.000)	(− 3.64, *P* < 0.000)	(− 2.57, *P* = 0.010)
Group 3	(3.21, *P* = 0.001)	(− 1.83, *P* = 0.067)	(− 3.65, *P* < 0.000)	(− 2.50, *P* = 0.012)
Between-group differences (Mann–Whitney *U* test)				
	Pain changes T1–T2 (standardized test statistics, *P* value)	Pain changes T2–T3 (standardized test statistics, *P* value)	Pain changes T3–T4 (standardized test statistics, *P* value)	Pain changes T1–T4 (standardized test statistics, *P* value)
Groups 1 and 2	(− 2.20, *P* = 0.030)	(− 3.09, *P* = 0.002)	(− 0.36, *P* = 0.728)	(− 1.22, *P* = 0.235)
Groups 1 and 3	(− 0.54, *P* = 0.607)	(− 1.12, *P* = 0.270)	(− 0.61, *P* = 0.550)	(− 1.48, *P* = 0.149)
Groups 2 and 3	(− 1.80, *P* = 0.080)	(− 1.65, *P* = 0.109)	(− 1.22, *P* = 0.246)	(− 0.737, *P* = 0.470)

*T1*, 1 h; *T2*, 24 h; *T3*, 48 h; *T4*, 1 week after insertion of interventional AW

Although all patients reported higher pain scores during the first 24 h of AW insertion (*P* < 0.01), it was the most in group 2. Less pain scores were reported in group 1 followed by group 3. Afterwards, pain scores started to reduce significantly in groups 2 compared to group 1 (*P* < 0.01). All groups reported similar pain scores after 48 h.

### Lower arch dimensions (Table 6)

**Table 6:** Means, SDs of lower AL, ICW, and IMW, Wilcoxon signed-rank (within-group differences) and Mann–Whitney *U* (between-group differences) standardized test statistics, and *P* values in the 3 studied groups

	Arch length (AL) (mm)		Intercanine width (ICW) (mm)		Intermolar width (IMW) (mm)	
	T1	T7	T1	T7	T1	T7
	Mean (SD)	Mean (SD)	Mean (SD)	Mean (SD)	Mean (SD)	Mean (SD)
Group 1 (0.017 × 0.025 SS)	24.83 (1.04)	27.00 (1.19)	26.19 (1.58)	28.06 (1.54)	44.11 (1.97)	45.94 (1.95)
Group 2 (0.019 × 0.025 SS)	24.35 (1.12)	27.24 (0.90)	26.71 (1.21)	29.15 (1.03)	46.79 (2.21)	49.05 (1.85)
Group 3 (0.021 × 0.025 TMA)	25.22 (1.11)	27.67 (1.03)	26.81 (1.20)	29.17 (1.30)	47.00 (2.85)	49.33 (3.08)
Within-group changes of AL, ICW, and IMW changes (mm) (related samples Wilcoxon signed-rank test)						
	AL changes T1–T7 (standardized test statistics, *P* value)		ICW changes T1–T7 (standardized test statistics, *P* value)		IMW changes T1–T7 (standardized test statistics, *P* value)	
Group 1	(3.84, *P* < 0.001)		(3.75, *P* < 0.000)		(3.67, *P* < 0.000)	
Group 2	(3.82, *P* < 0.001)		(3.70, *P* < 0.000)		(3.59, *P* < 0.000)	
Group 3	(3.81, *P* < 0.000)		(3.77, *P* < 0.000)		(3.78, *P* < 0.000)	
Between-group differences (Mann–Whitney ***U*** test)						
	AL changes (T1–T7) (standardized test statistics, *P* value)		ICW changes (T1–T7) (standardized test statistics, *P* value)		IMW changes (T1–T7) (standardized test statistics, *P* value)	
Groups 1 and 2	(− 3.28, *P* = 0.003)		(2.27, *P* = 0.029)		(− 1.48, *P* = 0.163)	
Groups 1 and 3	(− 1.40, *P* = 0.214)		(2.23, *P* = 0.290)		(− 1.66, *P* = 0.118)	
Groups 2 and 3	(− 1.93, *P* = 0.110)		(0.57, *P* = 0.613)		(− 0.05, *P* = 0.961)	

*T1*, before inserting interventional arch wires with reverse COS; *T7*, 6 months after inserting interventional arch wires with reverse COS

A significant increase in lower arch dimension was detected during leveling in all groups (*P* < 0.001). A total arch length increases of 2.17 mm, 2.88 mm, and 2.44 mm was achieved in groups 1, 2, and 3, respectively. The mean differences of 0.72 mm between groups 1 and 2 was significant at *P* < 0.01. During COS leveling, ICW increased 1.86 mm, 2.44 mm, and 2.36 mm in groups 1, 2, and 3, respectively. The mean difference of 0.58 mm between groups 1 and 2 was significant at *P* < 0.05. The IMW increase during leveling was similar in the 3 studied groups.

### Incisor and molar treatment changes (Table 7)

**Table 7:** Means, SDs, and Mann–Whitney *U* (between-group differences) standardized test statistics and *P* values in the 3 studied groups

Variables	Group 1	Group 2	Group 3	Groups 1 and 2	Groups 1 and 3	Groups 2 and 3
	Mean (SD)	Mean (SD)	Mean (SD)	Standardized statistics, *P* value	Standardized statistics, *P* value	Standardized statistics, *P* value
Incisor vertical movement (mm) (+ intrusion, − extrusion)	0.04 (0.63)	0.24 (0.42)	0.58 (0.60)	(− 0.85, *P* = 0.405)	(− 2.27, *P* = 0.022)	(− 2.14, *P* = 0.032)
Molar vertical movement (mm) (+ intrusion, − extrusion)	− 0.41 (0.81)	− 0.06 (0.48)	− 0.16 (0.51)	(− 1.39, *P* = 0.165)	(− 2.52, *P* = 0.011)	(− 2.09, *P* = 0.038)
Incisor crown forward movement (mm)	0.78 (0.32)	1.05 (0.54)	1.04 (0.32)	(− 1.86, *P* = 0.072)	(− 2.68, *P* = 0.008)	(− 0.66, *P* = 0.525)
Incisor root forward (mm)	0.32 (0.30)	0.59 (0.72)	0.45 (0.21)	(− 1.29, *P* = 0.219)	(− 1.88, *P* = 0.064)	(− 0.017, *P* = 0.987)
Molar crown (mm) (+ mesial, − Distal)	0.31 (0.93)	− 0.20 (1.19)	− 0.54 (0.89)	(− 1.61, *P* = 0.110)	(− 0.514, *P* = 0.628)	(− 1.70, *P* = 0.103)
Molar root (mm) (+ mesial, − distal)	0.06 (0.66)	0.03 (0.96)	0.06 (1.06)	(− 0.10, *P* = 0.935)	(− 0.37, *P* = 0.719)	(− 0.150, *P* = 0.883)

COS leveling was accompanied with lower incisor intrusion and lower first molar extrusion in all groups (*P* > 0.05). Lower incisor intrusion was more pronounced in group 3 compared to groups 1 and 2 (*P* < 0.05) and lower molar extrusion was more pronounced in group 1 compared to groups 2 and 3 (*P* < 0.05). More than 1 mm of lower incisor proclination was found in groups 2 and 3. A statistically significant difference was found between groups 1 and 3 only (*P* < 0.01).

## Discussion

Despite the perceived importance of the COS, there is little published research that compares arch leveling using various orthodontic AWs. Therefore, this investigation was carried out to investigate the efficacy of 3 different dimensions of continuous leveling AWs (0.017 × 0.025-inch SS, 0.019 × 0.025-inch SS, and 0.021 × 0.025-inch TMA for the correction of excessive COS in the mandibular arch and to report on lower incisors’ EARR, incisor intrusion, pain scores, and arch dimensional changes associated with leveling COS using the above AWs.

It has been reported that COS leveling differs between low and high angle groups. Rozzi et al. [18] found that leveling of the COS in low maxillary/mandibular (MM) angle group occurs through buccal movement and intrusion of the mandibular incisors, while in high MM angle subjects, it occurs through extrusion and uprighting of the posterior teeth. Furthermore, greater masticatory muscle activity and increased masticatory efficiency were reported in brachyfacial subjects [21], while less muscular activity was associated with high MM angle subjects. This increased muscular activity in short face subjects is usually accompanied with increased maximum occlusal bite force [22] which may affect posterior teeth in the vertical direction and limit their extrusion during leveling of COS. In the current study, low or normal MM angle subjects were included; none had high MM angle.

In the current study, although the 3 investigated AWs’ sizes were effective in leveling the COS within the trial period, 0.019 × 0.025-inch SS AW was the most effective. This might be explained by the high force expression produced by the thicker SS AW in group 2 as force expression depends on the amount of bracket slot-AW play, AW dimension, and AW stiffness [23]. The reported stiffness values for 0.019 × 0.25-inch SS AW are higher than those for 0.017 × 0.025-inch SS AW and 0.021 × 0.025-inch TMA AW which means that the amount of the delivered force in group 2 was higher compared to the other 2 groups [24]. In group 3, the less bracket slot-AW play did not compensate for the reduced AW stiffness and COS reduction was less in group 3 compared to group 2. In group 1, COS leveling was the least among the groups. The relatively larger slot-AW play associated with the smaller 0.017 × 0.025-inch SS AW may have produced less leveling force in group 1 [25]. It has been stated that SS AWs have a high value for strength and stiffness, while TMA generates produce gentle forces and deliver approximately half the force of SS AWs [25].

As the lower incisors in the current study were initially retroclined, the interventional AWs were not cinched back during COS leveling which might explain the increased arch dimensions. However, no significant axial inclination changes of the lower incisors have been reported after cinching the AW during treatment [26].

In the current study, 4 mm of COS leveling was accompanied by only 4° of lower incisor proclination. This was in contrary to Pandis et al. [27] who suggested that for every 1 mm of COS leveling, the lower incisors were proclined by 4°. In their study [27], flattening of COS was achieved without arch width increase, while in the current study, COS was leveled by both lower incisor proclination and increased arch width. The increased arch width during COS leveling provided the space needed to level COS and reduced lower incisor proclination.

Although less lower incisor proclination in group 3 might be anticipated due to less bracket slot-AW play, this was not the case in this investigation. Lower incisor proclination might have occurred as the intrusive force application by reverse COS is anterior to the center of resistance [28]. This finding is in line with others who suggested that excessive incisor tipping may result from high intrusive forces at the incisor brackets creating tipping moments on each incisor and that could happen with increase AW dimensions [29].

In addition to lower incisor proclination, COS was leveled by lower incisor intrusion. Incisor intrusion was more pronounced in group 3. TMAAW resulted in more intrusion compared to SS AWs (0.04 mm, 0.24 mm, and 0.58 mm in groups 1, 2, and 3, respectively). The less play between AW and bracket slot may have resulted in a more vertically directed forces allowing for more intrusion in groups 2 and 3 compared to group 1 regardless of the amount of intrusion forces exerted by each AW [30].

In the current study, lower molar extrusion accompanied COS leveling. Molar extrusion was found in group 1 (0.41 mm) compared to groups 2 and 3 (0.06 mm and 0.016 mm, respectively). This could be attributed to less molar axial control in that group due to the more AW and bracket slot play. In addition, this may have resulted from the expression of the built-in molar attachment tip back (6°) in most patients [6]. However, more tip back could have happened in the appliance prescription when using reverse COS AWs. The current finding was in agreement with Rozzi et al. [18] who suggested that COS leveling was accomplished by relative extrusion of the premolars, and buccal movement, uprighting, and extrusion of the mandibular molars.

Molar mesial root movement was noticed in all studied groups with no significant difference. This was in agreement with Clifford et al. [31] who stated that correcting accentuated COS happened in all cases mostly due to the mesial movement of the molar roots.

In general, EARR does not exceed 2 mm during orthodontic treatment [32]. The degree, frequency, and type of force applied have been linked to EARR [33]. In the current study, the amount of EARR was minimal and clinically insignificant (< 1 mm) in all groups. This finding was in agreement with a recent systematic review and meta-analysis to evaluate root resorption following orthodontic intrusion. They concluded that root resorption of less than 1 mm is expected after intrusion mechanics and that the amount of resorption is within the acceptable limits for clinical implication [34]. On the other hand, other previous studies suggested that the degree of root resorption increases with intrusion, especially in single-rooted teeth [35, 36].

It has also been suggested that heavier forces aggravate root resorption. In the current study, all incisors experienced EARR of less than 1 mm irrespective of force level. This was in agreement with Akl et al. [37] who concluded that root resorption usually occurs in association with orthodontic intrusion irrespective of the magnitude of the intrusive forces. On the other hand, other studies [11, 38] reported a correlation between increased initial force levels and increased root resorption. Increasing AW dimension means increased force level which was also linked to increase in the risk of root resorption [11].

In this study, orthodontic pain reached its peak level after 24 h of interventional AW insertion. Less pain was perceived in the 0.017 × 0.025-inch SS group, followed by the 0.021 × 0.025-inch TMA group and 0.019 × 0.025-inch SS group. Although force level in the 3 investigated AW groups were not measured in the current study, it is well accepted that force level increases with the increase in AW dimensions and stiffness [17]. This finding was in agreement with those who reported a greater pain intensity when higher forces were used at 24 h [39]. On the other hand, other studies found no statistically significant correlation between initial applied force levels and experienced pain [40].

In all studied groups, dental AL and width increased significantly after COS correction. It was suggested that AL increase could result from using continuous reverse COS AW to level COS [41]. Also, from space analysis point of view, COS is considered a crowding in the vertical dimension; as the amount of COS increases, the amount of vertical crowding increases [42]. Accordingly, in the non-extraction treatment, leveling COS will be on the expense of the arch dimension increase. Since the non-extraction treatment was adopted in this study, our findings were expected.

The largest ICW increase was found in the 0.019 × 0.025-inch SS group, whereas the largest IMW was detected in the 0.021 × 0.025-TMAAW group. This finding was supported by Gioka et al. [43] who stated that more torque was delivered with larger dimensions. Also, it was reported earlier that TMA AWs have a more significant transverse increase in the IMW than the ICW area [44].

The limitations of this study may include the following: root resorption was assessed in lower incisors only for 6 months after starting the intervention, although root resorption can start any time before, and high female/male ratio and both low and average vertical dimension subjects were included. The higher muscular activity in low angle subjects may have affected posterior teeth in the vertical direction and limited their extrusion during leveling of COS [21]. In addition, this study is a single-center study. It is worth mentioning that this study was designed to detect small effect size between the groups. This indicates that although the efficacy of the 3 AWs differed statistically, differences between the groups did not exceed 1 mm which is not clinically significant.

## Conclusions

- All investigated AWs were effective in leveling COS with the 0.019 × 0.025-inch SS AW being the most effective one.
- All investigated AWs produced minimal lower incisor EARR.
- COS leveling was achieved by lower incisors’ intrusion and proclination and by lower molar extrusion.
- 0.019 × 0.025-inch SS AW subjects reported the highest pain scores within 24 h of AW placement.
- After 48 h of AW placement, pain scores were similar in all groups.
- Dental arch dimensions were increased during the leveling of COS in all studied groups and were pronounced in 0.019 × 0.025-inch SS AW subjects.

## Generalizability

The results of this study indicated that the 3 investigated leveling AWs were effective for the leveling COS and at the same time safe on the roots of the lower anterior teeth.
